# A Question of Competing Rights, Priorities, and Principles: A Postscript to the Robert Wood Johnson Foundation Symposium on the Ethics of Childhood Obesity Policy

**Published:** 2011-08-15

**Authors:** Shiriki K. Kumanyika

**Affiliations:** Department of Biostatistics and Epidemiology, University of Pennsylvania School of Medicine. Dr Kumanyika is the founder and chair of AACORN.

Ethical arguments related to childhood obesity policies raised during the 2010 Robert Wood Johnson Foundation (RWJF) symposium (1-7) sharpen our awareness of complex and fundamental questions about the functions of society and how policies are defined and implemented to benefit different entities. As solutions unfold, winners and losers will emerge, just as winners and losers emerge when society fails to find solutions and keeps the status quo, which is unacceptable. Even if child and adolescent obesity rates now leveled off, the high prevalence would have far-reaching, adverse effects on the health and fitness of current and future generations and, therefore, on the economic and social health of the entire society (3).

What winning the battle against childhood obesity means at both population and individual levels is captured in the goal statement of an Institute of Medicine (IOM) committee that addressed childhood obesity prevention (Box) (8). In addition to the goals that are specific to body weight, the committee also highlighted goals related to children's healthful eating and physical activity patterns and achieving appropriate physical, psychological, and cognitive growth and development among children. These positive goals are beyond those that relate to avoiding adverse physical, emotional, and social health outcomes for obese children. They relate to fostering overall health and well-being, rather than only preventing specific diseases. The IOM committee's vision and the pathway to achieving it, as outlined in their examples of intermediate goals (Box), continue to be endorsed and elaborated on by other organizations, including RWJF (9). Certain papers in the RWJF symposium focused on policies that address the intermediate goals of changing institutional environments and food advertising practices to promote energy balance and making options for healthful foods and physical activity more routine and readily available (1,2,6).

**Box.** Goals of Obesity Prevention in Children and Youth

The goal of obesity prevention in children and youth is to create, through directed social change, an environmental-behavioral synergy that promotes: Reduction in the incidence of childhood and adolescent obesity Reduction in the prevalence of childhood and adolescent obesity Reduction of mean population body mass index (BMI) Improvement in the proportion of children meeting the Dietary Guidelines for Americans Improvement in the proportion of children meeting physical activity guidelines Achieving physical, psychological, and cognitive growth and developmental goals For *individual* children and youth: A healthy weight trajectory, as defined by the Centers for Disease Control and Prevention BMI charts A healthful diet (quality and quantity) Appropriate amounts and types of physical activity Achieving physical, psychosocial, and cognitive growth and developmental goals Because it may take a number of years to achieve and sustain these goals, intermediate goals are needed to assess progress toward reduction of obesity through policy and system changes. Examples include: Increased number of children who safely walk and bicycle to school Improved access to and affordability of fruits and vegetables for low-income populations Increased availability and use of community recreational facilities Increased opportunities for play and physical activity Increased number of new industry products and advertising messages that promote energy balance at a healthy weight Increased availability and affordability of healthful foods and beverages at supermarkets, grocery stores, and farmers' markets located in walking distance of the communities they serve Changes in institutional and environmental policies that promote energy balance Reprinted with permission from: Koplan JP, Liverman CT, Kraak VI, editors; Committee on Prevention of Obesity in Children and Youth. Preventing childhood obesity: health in the balance. Washington (DC): National Academies Press; 2005.

The IOM committee's vision relied on the societal obligation to protect and nurture children, thus emphasizing the obesity-promoting aspects of children's environments and the inability of children to control or adequately fend for themselves in such environments. Rights are mentioned in relation to corporate rights when referring to the debate regarding commercial free speech (eg, applying the First Amendment right of free speech to commercial entities) and banning advertising to children (8). Nonsmokers' rights are mentioned in an appendix that describes key elements of the social movement to reduce cigarette smoking (8). However, a child rights approach — the right of a child to grow and develop in an environment that promotes a healthy weight and healthy overall development — is not explicit.

The question of rights was explicit throughout the RWJF symposium (eg, including the concerns regarding rights of corporations to commercial free speech as they affect food advertising [2]). Other presentations addressed the rights of children in educational settings to options for healthful food and adequate physical activity (1,6); parental rights and responsibilities in protecting their children from harmful circumstances (1,2,5); ensuring the rights of obese children to protection from bias and stigmatization (7); and equal rights for all children, including those from ethnic minority populations or those from low-income families who depend on free and reduced-price school meals, as well as children with physical or mental disabilities or special health care needs (1,2,4-7). The inescapable conclusion is that policies that give priority to key ethical principles supporting the rights of children to grow and develop in healthy environments are essential to resolving the childhood obesity epidemic in ways that are acceptable and sustainable from a societal perspective.

The child rights approach to justifying child protective obesity policies is appealing and well-grounded from an ethical perspective, drawing on the broader concept of ensuring child welfare (10) and, more fundamentally, on global human rights principles — the Universal Declaration of Human Rights (11), the Declaration of the Rights of the Child (12), and the subsequent convention on the rights of the child as a legally binding treaty for ratifying countries (11,12). A rights-based approach has been specifically invoked as a principle supporting statutory actions to regulate the marketing of unhealthful foods and beverages to children (13). This approach presumably elevates the rights of children to a level that supersedes potentially conflicting rights claimed by food marketers. However, the concept that the rights of children will take precedence over the rights of others may be more idealistic than practical. Entities that stand to lose (ie, those that perceive an infringement on their rights) may not readily allow the rights of children or parents to supersede their rights. The question of power is inevitable. That is, operationally, having rights may be less important than having the power (collective and individual agency) to exercise one's rights or obtain one's entitled protections.

Ethical principles should be leveraged to justify interventions on behalf of children's rights, when applicable, and in opposition to entities such as corporations (Figure). In the policy arena, children, parents, and certain populations are considered vulnerable (ie, requiring societal protection) and will lose unless those with responsibility for tipping the scales in favor of the less powerful can be effective. Ethical principles are only that, principles. Societal outcomes are determined by how these principles become priorities and are made operational through power dynamics. What priorities are assigned to protecting the rights of different entities depends on societal attitudes. Decisions regarding who wins or loses must be justified and made palatable in the context of broader societal values and norms. The term *vulnerable populations* highlights the complexity of this concept (14) because the term implies a lack of power, which is logical when applied to children but less so when applied to their parents or to any other adults (eg, adults of an ethnic minority or low-income populations). Political opinions regarding the responsibility of people to use their agency individually are often translated into arguments and interventions that blame the victim (15). Therefore, considering the concept of vulnerability, when attributed to a group of adults, is also political and can imply a lack of competence or a lack of agency (eg, to make rational choices about what foods to buy for one's children or for the family). A competent-consumer argument is used to counter arguments for controls on targeted advertising (16). This approach denies the interaction between environment and behavior as well as the emotional rather than cognitive targets of modern marketing (2). It also leaves advocates in the odd position of having to argue that protected groups are incompetent on a certain level. Consequently, taking a child rights and even a civil rights perspective provides a more principled and less paternalistic argument by clarifying that concerns of power and resources are often more relevant than those of competence.

**Figure.**
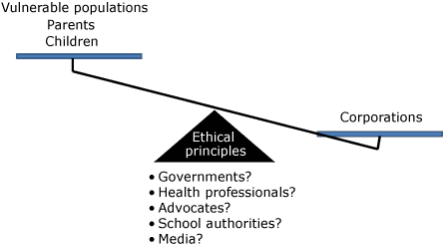
Ethical principles support the responsibility and ability of other societal entities to protect the rights of children, parents, and vulnerable populations more generally to a health-promoting (eg, non–obesity-promoting) environment in a context when these rights conflict with rights assigned to corporations that have more power and resources to defend their rights. To achieve balance requires alignment of diverse public and private attitudes regarding the most effective roles for governments and school authorities as well as eliminating stigmatization of people who are obese.

Discussing rights is simpler when the main topic is hunger. At the extremes of deprivation, attitudes more readily shift toward acceptance of the societal (ie, governmental) obligation to protect the less powerful and under-resourced segments of the population. Hungry people may evoke sympathy. When hungry people are fed, everyone wins — including corporations whose business is to sell food. In contrast, obesity is associated with having, and eating, too much food. Obese people do not evoke sympathy. In fact, people who are obese — and, for different reasons, members of ethnic or other demographic groups that have higher-than-average prevalence of obesity — are stigmatized and often blamed for their circumstances (7). Rights-based approaches are essential to ensure that children and parents, particularly children and parents among ethnic and social groups at high risk, are provided a fighting chance for sufficient leverage in this power struggle.
